# Reviewer Acknowledgement 2012

**DOI:** 10.1186/1471-2474-14-80

**Published:** 2013-03-20

**Authors:** Tim Shipley

**Affiliations:** 1 BioMed Central, Floor 6, 236 Gray's Inn Road, London, WC1X 8HB, United Kingdom

## Abstract

**Contributing reviewers:**

The editors of BMC Musculoskeletal Disorders would like to thank all our reviewers who have contributed to the journal in Volume 12 (2012).

## 

Nouran Abaza

Egypt

Albiruni Ryan Abdul Razak

Canada

Jacob Ablin

Israel

Carlos Acebes

Spain

Ilana Ackerman

Australia

Raphael Adobor

Norway

Nera Agabiti

Italy

Eva Ageberg

Sweden

Rohit Aggarwal

United States of America

Saleem Akbar

Mauritius

Toshihiro Akisue

Japan

Toru Akiyama

Japan

Yeliz Akkuş

Turkey

Oras Alabas

United Kingdom

Steven Albert

United States of America

Evangelos Alexopoulos

Greece

Naglaa Ali Gadallah

Egypt

Kelli Allen

United States of America

Marie Alricsson

Sweden

Nasser Al-Shanti

United Kingdom

Farhan Alvi

United Kingdom

Stephen Alway

United States of America

Richard Amerling

United States of America

Tassos Anastassiades

Canada

Isabel Andia

Spain

Felix Angst

Switzerland

Naffis Anjarwalla

United Kingdom

Maria Antonopoulou

Greece

Mitsuhiro Aoki

Japan

Adri Apeldoorn

Netherlands

Rachel Archer

United Kingdom

Monique Ardon

Netherlands

Kristina Areskoug Josefsson

Sweden

Miranda Elaine Glynis Armstrong

United Kingdom

Marc Asher

United States of America

Per Aspenberg

Sweden

Philip Atherton

United Kingdom

Henry Dushan Edward Atkinson

United Kingdom

Francisco Ayala

Spain

Charles Ayekoloye

United Kingdom

Olufemi Ayeni

Canada

Umesh Badrising

Netherlands

Yidong Bai

United States of America

Athan Baillet

France

Joshua Baker

United States of America

Federico Balagué

Switzerland

Maurice Balke

Germany

Manish Bansal

United Kingdom

Xenofon Baraliakos

Germany

Kamil Barbour

United States of America

Ann Barr

United States of America

Joaquin Barrios

United States of America

Else Marie Bartels

Denmark

Paul Beattie

United States of America

Ismail Bejia

Tunisia

Robert Bennett

United States of America

Enrico Benvenuti

Italy

Svante Berg

Sweden

Sasha Bernatsky

Canada

Boris Bershadsky

United States of America

Débora Bevilaqua-Grossi

Brazil

Ingo Beyer

Belgium

Mohit Bhandari

Canada

Bianca Bignotti

Italy

Amaia Bilbao

Spain

Bernd Bittersohl

Germany

Jakob Bjorner

Denmark

Sabine Blaschke

Germany

Dana Bliuc

Australia

Richard Bohannon

United States of America

Megan Bohensky

Australia

Alicja Joanna Bojan

Sweden

Nils Bömer

Netherlands

Roberta Bonfiglioli

Italy

Amy Bonomi

United States of America

Cecile RL Boot

Netherlands

Annemieke Boot

Netherlands

Lars Borgquist

Sweden

Julitta Boschman

Netherlands

Geoff Bostick

Canada

Mark Boswell

United States of America

Bronek Boszczyk

United Kingdom

Roger Bouillon

Belgium

Catherine Bowen

United Kingdom

Elizabeth Bradley

United States of America

Ivan Brenkel

United Kingdom

Peter Briggs

United Kingdom

Karen Briggs

United States of America

Karine Briot

France

Kim Brixen

Denmark

Matthew Brodie

Australia

Gert Bronfort

United States of America

Cathleen Brown

United States of America

Jens Ivar Brox

Norway

Olivier Bruyere

Belgium

Adam Bryant

Australia

Alex Burdorf

Netherlands

Paul Burgers

Netherlands

Rolf Burghardt

Germany

Hillevi Busch

Sweden

Cristina Cabral

Brazil

Mindy Cairns

United Kingdom

Jerrilyn Cambron

United States of America

Paul Campbell

United Kingdom

Carol Cancelliere

Canada

Beatriz Carames Perez

United States of America

Leah Carreon

United States of America

Emanuela Castelnuovo

United Kingdom

Filippo Castoldi

Italy

Fulvia Ceccarelli

Italy

Christine Cedraschi

Switzerland

Nicola Chaloner

United Kingdom

Priyanka Chandratre

United Kingdom

Ning-hung Chen

Taiwan

Weng-Pin Chen

Taiwan

Jack Cheng

Hong Kong

Linda Chesterton

United Kingdom

Hector Chinoy

United Kingdom

Sung-il Cho

Korea, South

Sungkun Cho

Korea, South

Saima Chohan

United States of America

Chung-Tei Chou

Taiwan

Flavia Cicuttini

Australia

Berta Cillero Pastor

Spain

Jean-Luc Clement

France

Pieter Coenen

Netherlands

David Coggon

United Kingdom

Paz Collado

Spain

Estelle Collin

Ireland

Paul Comfort

United Kingdom

Roger Componovo

United States of America

Philip Conaghan

United Kingdom

Christian Cook

United Kingdom

Glinda Cooper

United States of America

Olivier Cornu

Belgium

Salvatore Maria Corsello

Italy

Nelson Cortes

United States of America

Leonardo Costa

Brazil

Julie Côté

Canada

Francine Côté

France

David Cowan

United States of America

Michel D. Crema

Brazil

Albert Crenshaw

Sweden

Mervyn Cross

Australia

Tim Cundy

New Zealand

Caitriona Cunningham

Ireland

Bruno da Costa

Switzerland

Simon Dagenais

United States of America

Timothy Damron

United States of America

Cem Dane

Turkey

Nick Daneman

Canada

Michael Davie

United Kingdom

Eling D. de Bruin

Switzerland

F. Harald R De Man

Netherlands

Tommy de Windt

Netherlands

Marie Paule Defresne

Belgium

Francis Degache

Switzerland

Hans Degens

United Kingdom

Benjamin Deheshi

Canada

Eamonn Delahunt

Ireland

Giacomo Delle Rose

Italy

Xavier Demondion

France

Alasdair Dempsey

Australia

Kevin Deschamps

Belgium

Jules Desmeules

Switzerland

James DeVocht

United States of America

Rozalia Dimitriou

United Kingdom

Changhai Ding

Australia

Clermont Dionne

Canada

Dawn Doré

Australia

Michael Doschak

Canada

Jeffrey Driban

United States of America

Rosemary Dubbeldam

Germany

Lynsey Duffell

United Kingdom

Kate Dunn

United Kingdom

Ivan Leonardo Duque

Colombia

Isabelle Durand-Zaleski

France

Christian Duval

Canada

Jonathan Dyke

United States of America

Patrick Edery

France

Turgay Efe

Germany

Ulrich T. Egle

Germany

Eric Eils

Germany

Harald Ekedahl

Swaziland

Maha El Gaafary

Egypt

Abdellah El Maghraoui

Morocco

Achim Elfering

Switzerland

Bilal Farouk El-Zayat

Germany

Devakar Epari

Australia

Mehmet Erdil

Turkey

Pierre Ernst

Canada

Birgitte Espehaug

Norway

Deniz Evcik

Turkey

William Eward

United States of America

Ilker Eyupoglu

Germany

Jeremy Fairbank

United Kingdom

Francis Farhadi

United States of America

Rene Fejer

Denmark

Anna Ferguson

United Kingdom

Peter Ferguson

Canada

Jose Luis Fernandez

Spain

Benjamin Fernandez-Gutierrez

Spain

Manuela Ferreira

Australia

Georgios Filippou

Italy

Aharon Finestone

Israel

Milena Fini

Italy

Erica Fisci

Italy

Tore Fjalestad

Norway

Ladina Fliri

Switzerland

Thilo Floerkemeier

Germany

Casper Foldager

Denmark

Jafar Forghanizadeh

Iran

Nadine Foster

United Kingdom

Beth Fox

United States of America

Jochen Franke

Germany

Bruce Frankel

United States of America

Simon French

Australia

Helen French

Ireland

Jan C. Frich

Norway

Poul Frost

Denmark

Naoshi Fukui

Japan

Philipp Funovics

Austria

Takefumi Furuya

Japan

Fabio Galbusera

Germany

Jiri Gallo

Czech Republic

Rajiv Gandhi

Canada

Juerg Andreas Gasser

Switzerland

Marie Gdalevitch

Canada

Alan Geater

Thailand

Lars Gerhardsson

Sweden

Michelle Ghert

Canada

Alessio Giai Via

Italy

Peter Giannoudis

United Kingdom

Silvia Gianola

Italy

Richie Gill

United Kingdom

Justus Gille

Germany

Andrea Giusti

Italy

Manuela Glattacker

Germany

Mathias Glehr

Austria

Henning Glerup

Denmark

Débora Godoy-Izquierdo

Spain

Joerg Goldhahn

Switzerland

Stefano Gonnelli

Italy

Antonio Gonzalez

Spain

Jane Goodall

United Kingdom

Eric Goulet

Canada

Christopher Graham

United Kingdom

Florian Gras

Germany

Andrew Gray

New Zealand

Danka Grcevic

Croatia

Ed Greenfield

United States of America

Stefan Greiner

Germany

Phillip Gribble

United States of America

Theodoros B Grivas

Greece

Henning Gronbaek

Denmark

Thomas Gross

United States of America

Kenny Guex

Switzerland

Giulio Guido

Italy

Christine Guptill

Canada

Mitchell Haas

United States of America

Samer Habiba

Norway

Ehsan Habibi

Iran

Lisbet Haglund

Canada

Sebastien Hagmann

Germany

Toby Hall

Australia

Amanda Hall

Australia

Joseph Hamill

United States of America

Renzhi Han

United States of America

Michael Haneline

Malaysia

Kurt Hankenson

United States of America

Tariq Haqqi

United States of America

Vicki Harding

United Kingdom

Brendan Harley

United States of America

Anette Harris

Norway

Jan Hartvigsen

Denmark

Winfried Hauser

Germany

Cheryl Hawk

United States of America

John Hawse

United States of America

Daichi Hayashi

United States of America

Behzad Heidari

Iran

Yves Henchoz

Canada

Paul Hendrick

United Kingdom

Gordon Hendry

Australia

Peter Henningsen

Germany

Antonio Herrera

Spain

Lee Herrington

United Kingdom

Gregory Hicks

United States of America

Charlie Hicks-Little

United States of America

Atsushi Hijikata

Japan

Roger Hilfiker

Switzerland

Caroline Hing

United Kingdom

Toshiaki Hirose

Japan

Tina Histing

Germany

Anthony Hodsman

Canada

Victor Hoe

Malaysia

Caroline Hoemann

Canada

Sheilah Hogg-Johnson

Canada

Lexie Holliday

United States of America

J. Marcus Hollis

United States of America

Andreas Holtermann

Denmark

Robert Borden Hopkins

Canada

Manabu Hoshi

Japan

Petteri Hovi

Finland

Alan Hreljac

United States of America

Yong Hu

Hong Kong

Chun-Yuh Huang

United States of America

Ding Huanwen

China

Tricia Hubbard Turner

United States of America

Bionka Huisstede

Netherlands

Michael Anthony Hunt

Canada

David Hunter

Australia

David Hunter

United States of America

Vigdis Schnell Husby

Norway

Richard Hyde

United Kingdom

Masayuki Iki

Japan

Ross Iles

Australia

Brice Ilharreborde

France

Inaki Imaz

Spain

Joji Inamasu

Japan

Andreea Ioan

Netherlands

Ruxandra Ionescu

Romania

Ulrich Irlenbusch

Germany

Antoine Italiano

France

Kimieru Ito

Japan

Kenta Ito

Japan

Jun Iwamoto

Japan

A. Alex Jahangir

United States of America

Markus Due Jakobsen

Denmark

Chantal Janelle

Canada

Prawit Janwantanakul

Thailand

Mohamed Jarraya

United States of America

Barbara Jemec

United Kingdom

Chris Jensen

Denmark

Ching-Chuan Jiang

Taiwan

Clare Jinks

United Kingdom

Venerina Johnston

Australia

Morgan Jones

United States of America

Richard Jones

United Kingdom

Gabby Joseph

United States of America

Niels Gunnar Juel

Norway

Anne Juvani

Finland

Melissa Kacena

United States of America

Yuho Kadono

Japan

Ivo Kalajzic

United States of America

John Kalbfleisch

United States of America

Brita Kaltenbrunner Bernitz

Sweden

Steven Kamper

Australia

Lixin Kan

United States of America

Alexander Katalinic

Germany

Lesley Kay

United Kingdom

Cemal Kazimoglu

Turkey

Anne-Maree Keenan

United Kingdom

Joern Kekow

Germany

Anne Keller

Denmark

Gráinne Kelly

Ireland

Randall Keyser

United States of America

Thomas Johan Kibsgård

Norway

Jae-Do Kim

Korea, South

Klaus Kirketerp-Møller

Denmark

Koshi Kishimoto

Japan

Ingvild Kjeken

Norway

Christian Kleber

Germany

Travis Klein

Australia

Andre Klussmann

Germany

Patrick Knott

United States of America

Jih-Yang Ko

Taiwan

Chin-Chu Ko

Taiwan

Suleyman Koca

Turkey

Werner Kolb

Germany

Raghu Kolluri

United States of America

Elizaveta Kon

Italy

Alice Kongsted

Denmark

Kika Konstantinou

United Kingdom

Anastasios Korompilias

Greece

Tomasz Kotwicki

Poland

John Kovaleski

United States of America

Andreas Kranzl

Austria

Sabine Krause

Germany

Matthijs R. Krijnen

Netherlands

Ramaswamy Krishnan

United States of America

Vicki Kristman

Canada

Yutaka Kuroda

Japan

Markus Kuster

Australia

Alexandre Lädermann

Switzerland

Stefan Ladwig

Germany

George Yves Laflamme

Canada

Jerome Lafont

France

Kerstin Landin-Wilhelmsen

Sweden

Lisa Langsetmo

Canada

Brent Lanting

Canada

Malinee Laopaiboon

Thailand

Lasse J Lapidus

Sweden

A. Noelle Larson

United States of America

Maria Larsson

Sweden

Laura Laslett

Australia

Marissa Lassere

Australia

Thomas Läubli

Switzerland

Yocheved Laufer

Israel

Miranda Laurant

Netherlands

Henrik Hein Lauridsen

Denmark

Michael Lavagnino

United States of America

Stewart Leavitt

United States of America

Charlotte Leboeuf-Yde

Denmark

Florent Lebon

France

Miryoung Lee

United States of America

Yvonne Lee

United States of America

Gwo-Chin Lee

United States of America

Guang-hua Lei

China

Päivi Leino-Arjas

Finland

Andreas Leithner

Austria

Erik Lenguerrand

United Kingdom

Frankie Leung

Hong Kong

Ying Ying Leung

Singapore

Paul Lewis

United States of America

Guoan Li

United States of America

Zheng Li

China

Nanxin Li

United States of America

Kang Li

United States of America

Yin-Ming Li

Taiwan

Bin Li

China

Yuan LI

United States of America

Elisabeth Lie

Norway

Ivan Lin

Australia

Xuhui Liu

United States of America

Yi Liu

China

Therese Ljungquist

Sweden

JiaHsuan Lo

United States of America

Grace Lo

United States of America

David Logerstedt

United States of America

Heinz Lohrer

Germany

Carl Lombard

South Africa

Eija Lönnroos

Finland

Maria Lopez-Armada

Spain

Asuncion Lopez-Calderon

Spain

Rik Lories

Belgium

John Loughlin

United Kingdom

Quinette Louw

South Africa

Estibaliz Loza

Spain

Juan V. Luciano

Spain

Jolanda Luime

Netherlands

Teija Lund

Finland

Zong-Ping Luo

China

Hannu Luomajoki

Switzerland

Joy MacDermid

Canada

David MacDonald

Australia

Dawn Mackey

United States of America

Pascal Madeleine

Denmark

Henning Madry

Germany

Nicola Maffulli

United Kingdom

Paolo Magni

Italy

Chris Maher

Australia

Andrea Maier

Netherlands

Evelina Maines

Italy

Tokifumi Majima

Japan

Keijo Makela

Finland

Davide Malatesta

Switzerland

Konstantinos Malizos

Greece

Peter Mandl

Austria

Sokichi Maniwa

Japan

Cindy Mann

United Kingdom

Claus Manniche

Denmark

Minna Mannikko

Finland

Pekka Mäntyselkä

Finland

Thomas Maribo

Denmark

Bizzini Mario

Switzerland

Laurence Marshman

Australia

Toru Maruyama

Japan

Nicola Massy-Westropp

Australia

Koichi Masuda

United States of America

Morio Matsumoto

Japan

Takashi Matsushita

Japan

Stephen May

United Kingdom

Masood Mazaheri

Iran

Saeideh Mazloomzadeh

Iran

James McAuley

Australia

David McBride

New Zealand

Christopher McCarthy

United Kingdom

Ulrik Mccarthy Persson

Ireland

Damian McClelland

United Kingdom

Lance McCracken

United Kingdom

Christina McCrae

United States of America

Suzanne McDonough

United Kingdom

C. Wayne McIlwraith

United States of America

Andrew McLachlan

Australia

Toni McLaurin

United States of America

Francesc Medina-Mirapeix

Spain

Geert Meermans

Belgium

Morteza Meftah

United States of America

Christopher Mendias

United States of America

Anne Marit Mengshoel

Norway

Gabriele Meyer

Germany

Sofia Michopoulou

United Kingdom

Harald Miedema

Netherlands

Alberto Migliore

Italy

Anna Mika

Poland

Lone Ramer Mikkelsen

Denmark

Steve Milanese

Australia

Petar Milovanovic

Serbia

Stefan Milz

Germany

Marco Alessandro Minetto

Italy

Yukihide Minoda

Japan

David Mitton

France

Takeshi Miyamoto

Japan

Keita Miyanishi

Japan

Ali Mobasheri

United Kingdom

Hitesh Modi

Korea, South

Nicola Mok

Hong Kong

Maria José Molina-Garrido

Spain

Niamh Moloney

Australia

Mauro Mondelli

Italy

David Monroe

United States of America

Michael A. Mont

United States of America

Ali Montazeri

Iran

Dirk Jan Moojen

Canada

Andrew Moore

United Kingdom

Babak Moradi

Germany

David Morgenroth

United States of America

Paul Jarle Mork

Norway

Tone Morken

Norway

Isaacl Moss

United States of America

Goro Motomura

Japan

Thomas Mroz

United States of America

Christian-Andreas Mueller

Germany

Michiel Mulier

Belgium

Dirk Müller

Germany

Shigeyuki Muraki

Japan

Chris Murphy

United Kingdom

Takeo Nagura

Japan

Toshiyasu Nakamura

Japan

Hiroaki Nakamura

Japan

Annu Näkki

Finland

Surena Namdari

United States of America

Akihide Nampei

Japan

Madhumita Nandi

India

Raghu Natarajan

United States of America

Rahul Nath

United States of America

Bård Natvig

Norway

Tanja Nauck

Germany

Justine Naylor

Australia

Guo-Xin Ni

China

Helen Nicholson

New Zealand

Yasuo Niki

Japan

Gertrud Nilsson

Sweden

Satoru Noguchi

Japan

Peter Nolte

Netherlands

Angela Notarnicola

Italy

Christine Novak

Canada

Montserrat Núñez

Spain

Michael T. Nurmohamed

Netherlands

Osondu Nwangwu

United Kingdom

Neil O’Connell

United Kingdom

John O’Donnell

Australia

Sarah Ohrndorf

Germany

Naomi Oizumi

Japan

Hiroyuki Oka

Japan

Francesco Oliva

Italy

Ignazio Olivieri

Italy

Elizabeth Olmsted-Davis

United States of America

Gunnar Olsson

Sweden

Bie Nio Ong

United Kingdom

Patrick Orth

Germany

Csaba Ortutay

Finland

Manathip Osiri

Thailand

Georg Osterhoff

Switzerland

Elisa Pala

Italy

Jerzy Palka

Poland

Norbert Pallua

Germany

George Panayiotakis

Greece

Evangelos Pappas

United States of America

Jongbae Park

United States of America

Jong-Beom Park

Korea, South

Dora Pascual-Salcedo

Spain

Amanda Patrick

United States of America

Ingris Pelaez-Ballestas

Mexico

Jean-Pierre Pelletier

Canada

Matt Pelletier

Australia

Praneet Pensri

Thailand

Cristina Perez

Spain

Muthu Periasamy

United States of America

Stephen Perle

United States of America

Carlo Perricone

Italy

Meredith Perry

New Zealand

Shannon Petersen

United States of America

Brad Petrisor

Canada

Danielle Petruccelli

Canada

Robert Pflugmacher

Germany

Susan Picavet

Netherlands

Wolfgang Pichler

Austria

Karin Pieber

Austria

Sérgio Piedade

Brazil

Fabio Pigozzi

Italy

Peter Pilot

Netherlands

Giulio Pioli

Italy

Pertti Pirttiniemi

Finland

Wirawit Piyamongkol

Thailand

Anna Plaas

United States of America

Nicolas Place

Switzerland

Timothy Platts-Mills

United States of America

Stéphane Poitras

Canada

Henry Pollard

Australia

Richard Pope

United States of America

Raewyn Poulsen

United Kingdom

Yong-Hao Pua

Singapore

Enriqueta Pujol-Ribera

Spain

Lia Pulsatelli

Italy

Weiping Qin

United States of America

Yong Qiu

China

Jose Manuel Quesada Gomez

Spain

Nasir A. Quraishi

United Kingdom

Lars Rackwitz

Germany

Hannah Radley-Crabb

Australia

Jayaprakash Raman

Canada

Sofia Ramiro

Netherlands

Claus Rasmussen

Denmark

Arif Razak

United Kingdom

Pascal Reboul

France

Ignacio Rego-Perez

Spain

Ines Reichert

United Kingdom

Max Reijman

Netherlands

Inge Reininga

Netherlands

Silje Endresen Reme

United States of America

Peter Rhee

United States of America

Jose A. Riancho

Spain

Eva Ribom

Sweden

Dan Riddle

United States of America

Johan Rietman

Netherlands

Rolf Riise

Norway

Patrick Riley

United States of America

David Ring

United States of America

Bernhard Rintelen

Austria

Bill Ristevski

Canada

Greg Robertson

United Kingdom

Edward Roddy

United Kingdom

Ana Rodriguez Garcia

Spain

Kirsten Kaya Roessler

Denmark

Cecilia Rogmark

Sweden

Sattaya Rojanasthien

Thailand

Jorge A. Roman-Blas

United States of America

Evan Romas

Australia

Nahum Rosenberg

Israel

Dale Rublee

United States of America

Marina Rull

Mexico

Peter Rundquist

United States of America

Sarah Ryan

United Kingdom

Isabel Sacco

Brazil

Bahar Sadeghi Abdollahi

Iran

Amets Sáenz

Spain

Christelle Sanchez

Belgium

Tom Sanders

United Kingdom

Tormenta Sandro

Italy

Augusto Hasiak Santo

Brazil

Borja Sañudo

Spain

Hilla Sarig Bahat

Israel

Luca Saturnino

Italy

Judith Sautner

Austria

Matthias Schafroth

Netherlands

Mandy Scheermesser

Switzerland

Hanna Schell

Germany

Niels Schep

Netherlands

Elena Schiopu

United States of America

Marcus Schmitt-Sody

Germany

Michael Schnabel

Germany

Michael Schneider

United States of America

Vanessa Scholtes

Netherlands

Elisa Schrank

United States of America

Johannes Schröder

Germany

Ralf-Juergen Schroeder

Germany

Gundula Schulze-Tanzil

Germany

Carlo Alberto Scirè

Italy

Giorgio Scivoletto

Italy

John Scolaro

United States of America

Luca Maria Sconfienza

Italy

Luigia Scudeller

Italy

Jesús Seco

Spain

Timothy Sell

United States of America

Anne Grete Semb

Norway

Vladimir Senekovic

Slovenia

Raj Sengupta

United Kingdom

Olivier Seynnes

Norway

Roshan Shah

United States of America

Jay Shapiro

United States of America

William Shaw

United States of America

Liba Sheeran

United Kingdom

Charles Sheets

United States of America

Neil Sheth

United States of America

Michiel Siebelt

Netherlands

Herrick Siegel

United States of America

Frank Siemers

Germany

Siddhartha Sikdar

United States of America

Maureen Simmonds

United States of America

Kern Singh

United States of America

Rohit Singhal

United Kingdom

Patricia Sinnott

United States of America

Parco Siu

Hong Kong

Eva Skillgate

Sweden

Frank Skorpen

Norway

Gerard Slobogean

Canada

Tuulikki Sokka

Finland

Svetlana Solovieva

Finland

Guanbin Song

China

Bing Song

United Kingdom

Soon Song

United Kingdom

Jan Sorensen

Denmark

Hiroyuki Sorimachi

Japan

Gunter Spahn

Germany

Robert Spinner

United States of America

Dawn Stacey

Canada

Vincent Stadelmann

Switzerland

Philip Stahel

United States of America

Richard Stange

Germany

Tasha Stanton

Australia

David Stelzeneder

Austria

Dirk Stengel

Germany

Maria Stoenoiu

Belgium

Ian Stokes

United States of America

Johannes Strunk

Germany

Yulong Sun

China

Tiezheng Sun

China

Reijo Sund

Finland

Paul Sung

Korea, South

Akinobu Suzuki

Japan

Susanne Wulff Svendsen

Denmark

Jan A. Swinkels

Netherlands

Magnus Tägil

Sweden

Alberto Tagliafico

Italy

Beril Talim

Turkey

Moritz Tannast

Switzerland

Kimberly Templeton

United States of America

Anne Thackeray

United States of America

David Thomas

Australia

Bregje Thomassen

Netherlands

Stavros Thomopoulos

United States of America

Kristian Thorborg

Denmark

Andrew Thoreson

United States of America

Carsten Oliver Tibesku

Germany

Saket Tibrewal

United Kingdom

Tina Tina Duong

United States of America

Harukazu Tohyama

Japan

Fatih Tok

Turkey

Allan Toomingas

Sweden

Grant Townsend

Australia

Catherine Trask

Canada

Guglielmo Trovato

Italy

Anna Trulsson

Sweden

Anika Tsuchida

Netherlands

Yuan-Kun Tu

Taiwan

Gabrielle Tuijthof

Netherlands

Sven Tulner

Netherlands

Steve Tumilty

New Zealand

Tiraje Tuncer

Turkey

Robert Turcotte

Cayman Islands

Glen Turley

United Kingdom

Takafumi Ueda

Japan

Martin Underwood

United Kingdom

P Nithin Unnikrishnan

United Kingdom

Peter Vaes

Belgium

Sander Van Assen

Netherlands

Fons Van de Loo

Netherlands

Joop Van den Bergh

Netherlands

Wilbert Van den Hout

Netherlands

Josien Van den Noort

Netherlands

Wim Van den Noortgate

Belgium

Huub Van der Heide

Netherlands

Olav Van der Jagt

Netherlands

Peter Van der Kraan

Netherlands

Jan Van der Meulen

United Kingdom

Walter Van der Weegen

Netherlands

Philip Van der Wees

Netherlands

Gary Van Guilder

United States of America

Hans-Peter Van Jonbergenan Jonbergen

Netherlands

Jakob Van Oldenrijk

Netherlands

Luc Van den Bossche

Belgium

Patrick Vavken

United States of America

Darcy Vavrek

United States of America

Jeanine Verbunt

Netherlands

Howard Vernon

Canada

Peter Vestergaard

Denmark

Wolfgang Viechtbauer

Netherlands

Marijn Vis

Netherlands

Jetze Visser

Netherlands

Tuomo Visuri

Finland

Annemarie Vlaar

Netherlands

Violeta Maria Vlad

Romania

Brigitte Von Rechenberg

Switzerland

Sunil Wadhwa

United States of America

Morten Wærsted

Norway

Philippe Wagner

Sweden

Brian Walitt

United States of America

Karen Walker-Bone

United Kingdom

Jack Wall

Australia

Yuanyuan Wang

Australia

Tim Watson

United Kingdom

Connie Weaver

United States of America

Christian Wedemeyer

Germany

Lars Weidenhielm

Sweden

Paul Weinhold

United States of America

Stephanie Shifra Weinreich

Netherlands

Kurt Weiss

United States of America

William Welch

United States of America

Claire Yvette Wenham

United Kingdom

Kerstin Wentz

Sweden

Sarah West

Canada

Michael Whitehouse

United Kingdom

Rod Whiteley

Qatar

Garth Whiteside

United States of America

Samuel Whittle

Australia

Erik Wikstrom

United States of America

Britt Wildemann

Germany

Lukas Wildi

Switzerland

Arne Wilharm

Germany

Ross Wilkie

United Kingdom

Anita Williams

United Kingdom

Frances Williams

United Kingdom

Dale Williams

Canada

Esther Williamson

United Kingdom

Fiona Wilson

Ireland

Patarawan Woratanarat

Thailand

Ximena Wortsman

Chile

Edward Wright

United States of America

Gwenllian Wynne-Jones

United Kingdom

Tatsuo Yamamoto

Japan

Takuaki Yamamoto

Japan

Takuma Yamasaki

Japan

Simon Yeung

Hong Kong

Erlangga Yusuf

Netherlands

Ishimoto Yuyu

Japan

Fabio Zaina

Italy

Paul Zalzal

Canada

Boris Zelle

United States of America

Weiya Zhang

United Kingdom

Xiangsheng Zhang

China

Wei Zhang

United States of America

Naiquan Zheng

United States of America

Xiaodong Zhou

United States of America

Zhen An Zhu

China

Christoph Zilkens

Germany

Gerald Zimmermann

Germany

Lorne Zinman

Canada

Francesco Zulian

Italy

## Pre-publication history

The pre-publication history for this paper can be accessed here:

http://www.biomedcentral.com/1471-2474/14/80/prepub

